# Reducing charge noise in quantum dots by using thin silicon quantum wells

**DOI:** 10.1038/s41467-023-36951-w

**Published:** 2023-03-13

**Authors:** Brian Paquelet Wuetz, Davide Degli Esposti, Anne-Marije J. Zwerver, Sergey V. Amitonov, Marc Botifoll, Jordi Arbiol, Amir Sammak, Lieven M. K. Vandersypen, Maximilian Russ, Giordano Scappucci

**Affiliations:** 1 QuTech and Kavli Institute of Nanoscience, Delft University of Technology, PO Box 5046, 2600 GA Delft, The Netherlands; 2 QuTech and Netherlands Organisation for Applied Scientific Research (TNO), Delft, The Netherlands; 3 Catalan Institute of Nanoscience and Nanotechnology (ICN2), CSIC and BIST, Campus UAB, Bellaterra, 08193 Barcelona, Catalonia Spain; 4 ICREA, Pg. Lluís Companys 23, 08020 Barcelona, Catalonia Spain

**Keywords:** Quantum dots, Spintronics

## Abstract

Charge noise in the host semiconductor degrades the performance of spin-qubits and poses an obstacle to control large quantum processors. However, it is challenging to engineer the heterogeneous material stack of gate-defined quantum dots to improve charge noise systematically. Here, we address the semiconductor-dielectric interface and the buried quantum well of a ^28^Si/SiGe heterostructure and show the connection between charge noise, measured locally in quantum dots, and global disorder in the host semiconductor, measured with macroscopic Hall bars. In 5 nm thick ^28^Si quantum wells, we find that improvements in the scattering properties and uniformity of the two-dimensional electron gas over a 100 mm wafer correspond to a significant reduction in charge noise, with a minimum value of 0.29 ± 0.02 μeV/Hz^½^ at 1 Hz averaged over several quantum dots. We extrapolate the measured charge noise to simulated dephasing times to CZ-gate fidelities that improve nearly one order of magnitude. These results point to a clean and quiet crystalline environment for integrating long-lived and high-fidelity spin qubits into a larger system.

## Abstract (web summary)

Charge noise degrades the performance of spin qubits hindering scalability. Here the authors engineer the heterogeneous material stack in ^28^Si/SiGe gate-defined quantum dots, to improve the scattering properties and to reduce charge noise.

## Introduction

Spin-qubits in silicon quantum dots are a promising platform for building a scalable quantum processor because they have a small footprint^1^, long coherence times^2,3^, and are compatible with advanced semiconductor manufacturing^4^. Furthermore, rudimentary quantum algorithms have been executed^5^ and quantum logic at high-fidelity performed^6–9^. As the qubit count is increasing, with a six-qubit processor demonstrated^10^, significant steps have been taken to couple silicon spin qubits at a distance, via microwave photons or spin shuttling^11–16^, towards networked spin-qubit tiles^17^. However, electrical fluctuations associated with charge noise in the host semiconductor can decrease qubit readout and control fidelity^18^. Reducing charge noise independently of the device location on a wafer is pivotal to achieving the ubiquitous high-fidelity of quantum operations, within and across qubit tiles, necessary to execute more complex quantum algorithms.

Charge noise is commonly associated with two-level fluctuators (TLF)^19^ in the semiconductor host. In gated heterostructures with buried quantum wells, TLF may arise from impurities in several locations: within the quantum well, the semiconductor barrier, the semiconductor/dielectric interface, and the dielectrics layers above^20–26^. Furthermore, previous work on strained-Si MOSFETs^27–29^, with strained-Si channels deposited on SiGe strain relaxed buffers, has associated charge noise with dislocations arising from strain relaxation, either deep in the SiGe buffer or at the quantum well/buffer interface. Since these impurities and dislocations are randomly distributed over the wafer and are also a main scattering source for electron transport in buried quantum wells^30^, a holistic approach to materials engineering should be taken to address disorder in two-dimensional electron gases and charge noise in quantum dots.

In this work, we demonstrate thin quantum wells in ^28^Si/SiGe heterostructures with low and uniform charge noise, measured over several gate-defined quantum dot devices. By linking charge noise measurements to the scattering properties of the two-dimensional electron gas, we show that a quiet environment for quantum dots is obtained by improving the semiconductor/dielectric interface and the crystalline quality of the quantum well. We feed the measured charge noise into a theoretical model, benchmark the model against recent experimental results^6,10^, and predict that these optimized heterostructures may support long-lived and high-fidelity spin qubits.

## Results

### Description of ^28^Si/SiGe heterostructures

**Fig. 1:**
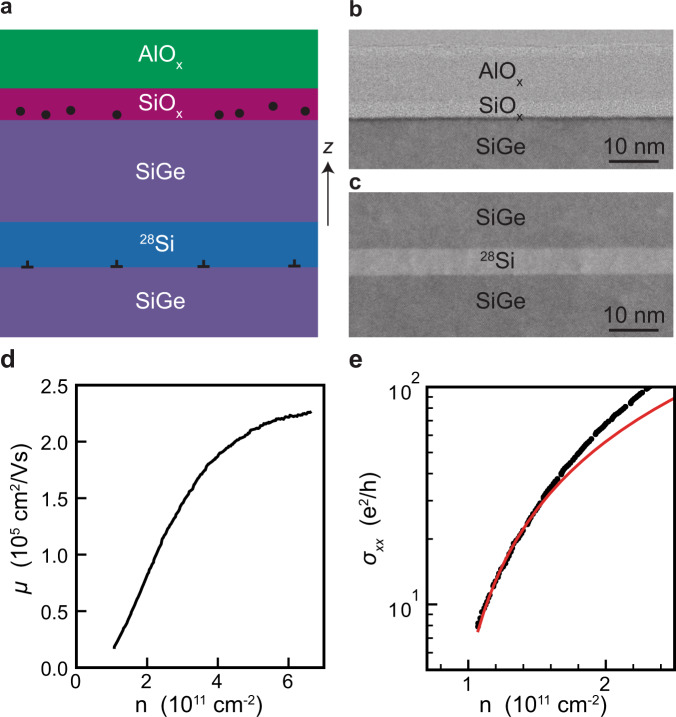
Material stack and heterostructure field effect transistor characterization. **a** Schematics of the ^28^Si/SiGe heterostructure and dielectric stack above. *z* indicates the heterostructure growth direction. Circles represent remote impurities at the semiconductor/dielectric interface and perpendicular symbols represent misfit dislocations that might arise at the quantum well/buffer interface due to strain relaxation. **b**, **c** BF-STEM images from heterostructure C highlighting the semiconductor/dielectric interface and the 5 nm thick ^28^Si quantum well, respectively. **d** Mobility *μ* and **e** conductivity *σ*_*xx*_ measured as a function of density *n* at a temperature of 1.6 K in a Hall bar H-FET from heterostructure C. The red curve in **e** is a fit to percolation theory.

Figure 1a illustrates the undoped ^28^Si/SiGe heterostructures, grown by reduced-pressure chemical vapor deposition, and the gate-stack above. From bottom to top, the material stack comprises a 100 mm Si substrate, a strain-relaxed SiGe buffer layer, a strained ^28^Si quantum well, a 30 nm thick SiGe barrier, a Si cap oxidized in air to form a SiO_*x*_ layer, an AlO_*x*_ layer formed by atomic layer deposition, and metallic gates. The SiGe layers above and below the quantum well have a Ge concentration of ≃0.3 (Methods).

We consider three ^28^Si/SiGe heterostructures (A, B, C) to improve, in sequence, the semiconductor/dielectric interface (from A to B) and the crystalline quality of the quantum well (from B to C). Heterostructure A has an ≃9 nm thick quantum well and is terminated with an epitaxial Si cap grown by dichlorosilane at 675 °C. This kind of heterostructure has already produced high performance spin-qubits^6,10,31^. Heterostructure B misses a final epitaxial Si cap but features an amorphous Si-rich layer obtained by exposing the SiGe barrier to dichlorosilane at 500 °C. Compared to A, heterostructure B supports a two-dimensional electron gas with enhanced and more uniform transport properties across a 100 mm wafer, owing to a more uniform SiO_*x*_ layer with less scattering centers^32^. Finally, we introduce here heterostructure C, having the same amorphous Si-rich termination as in heterostructure B, but a thinner quantum well of ≃5 nm (Supplementary Fig. 1). This is much thinner than the Matthews-Blakeslee critical thickness^33,34^, which is ≃10 nm^35^ for the relaxation of tensile Si on Si_0.7_Ge_0.3_ via the formation of misfit dislocation at the bottom interface of the quantum well. In light of recent morphological characterization by electron channeling constrast imaging of Si/SiGe heterostructures with similar quantum well thickness and SiGe chemical composition^36^, we expect misfit dislocation segments in heterostructure B because the quantum well approaches the Matthews-Blakeslee critical thickness. Due to the much thinner quantum well, instead, the epitaxial planes may adapt to the SiGe buffer much better in heterostructure C than in heterostructure B, meaning that misfit dislocations are, in principle, suppressed.

Figure 1b, c shows bright-field scanning transmission electron microscopy (BF-STEM) images from heterostructure C after fabrication of a Hall bar shaped heterostructure field effect transistors (H-FET). We observe a sharp SiGe/SiO_*x*_ semiconductor/dielectric interface (Fig. 1b), characterized by a minor Ge pile up (dark line) in line with ref. ^32^. The ≃5 nm thick quantum well (Fig. 1c, Supplementary Fig. 1) is uniform, has sharp interfaces to the nearby SiGe, and appears of high crystalline quality.

### Electrical characterization of heterostructure field effect transistors

We evaluate the scattering properties of the two-dimensional electron gases by wafer-scale electrical transport measured on Hall-bar shaped H-FETs operated in accumulation mode (Methods). For each heterostructure, multiple H-FETs over a wafer are measured in the same cool-down at a temperature of 1.7 K in refrigerators equipped with cryo-multiplexers^37^. Figure 1d, e shows typical mobility-density and conductivity-density curves for heterostructure C, from which we extract the mobility measured at high density (*n* = 6 × 10^11^ cm^−2^) and the percolation density (*n*_*p*_)^38^. The mobility rises steeply at low density due to progressive screening of scattering from remote impurities and flattens at higher density (*n* > 5 × 10^11^ cm^−2^), limited by scattering from impurities within or nearby the quantum well, for example uniform background charges, surface roughness, or crystalline defects such as threading or misfit dislocations^30,39^.

### Charge noise measurements in quantum dots

**Fig. 2:**
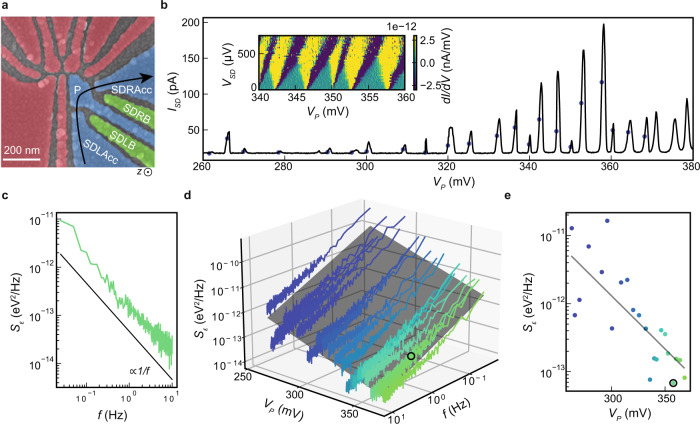
Quantum dots and charge noise measurements. **a** False colored SEM-image of a double quantum dot system with a nearby charge sensor. Charge noise is measured in the multi-electron quantum dot defined by accumulation gates SDLAcc and SDRAcc (blue), plunger P (blue), with the current going along the black arrow. In these experiments, the gates defining the double quantum dot (red) are used as screening gates. There is an additional global top gate (not shown) to facilitate charge accumulation when needed. **b** Source-drain current *I*_*SD*_ through a charge sensor device fabricated on heterostructure C against the plunger gate voltage *V*_*P*_. Colored dots mark the position of the flank of the Coulomb peak where charge noise measurements are performed. The inset shows Coulomb diamonds from the same device, plotted as the differential of the current *dI*/*dV* as a function of *V*_*P*_ and the source drain bias *V*_*SD*_. **c** Charge noise spectrum *S*_*ϵ*_ measured at the Coulomb peak at *V*_*P*_ ≃ 360.3 mV in **b** and extracted using the lever arm from the corresponding Coulomb diamond. The black trendline is proportional to 1/*f*. **d** *S*_*ϵ*_ for the same device in **b**, plotted in 3D as a function of *f* and *V*_*P*_. The dark gray plane is a fit through the datasets, i.e. the collection of noise spectra as in **c** measured at different *V*_*P*_ and each obtained using a unique lever arm from the corresponding Coulomb diamond. **e** Line cut through the data in **d** at *f* = 1 Hz, showing the experimental noise *S*_*ϵ*_ (colored dots) and fit (dark gray line). The black circled data point (also in **d**) marks the minimum charge noise measured for this specific device (*S*_*ϵ*,*min*_) at *f* = 1 Hz.

For charge noise measurements, we use devices comprising a double quantum dot and a charge sensor quantum dot nearby, illustrated in Fig. 2a. Using the same device design, two-qubit gates with fidelity above 99% were demonstrated^6^, silicon quantum circuits were controlled by CMOS-based cryogenic electronics^31^, and energy splittings in ^28^Si/SiGe heterostructures were studied with statistical significance^40^.

Here, we electrostatically define a multi-electron quantum dot in the charge sensor by applying gate voltages to the accumulation gates SDRAcc and SDLAcc, the barriers SDLB and SDRB, and the plunger gate P. All other gates (red in Fig. 2a) are set to 0 V for measurements of heterostructure B and C, whereas they are positively biased in heterostructure A to facilitate charge accumulation in the sensor (Methods). Figure 2b shows typical Coulomb blockade oscillations of the source-drain current *I*_*SD*_ for a charge sensor from heterostructure C measured at a dilution refrigerator base temperature of 50 mK. We follow the same tune-up procedure (Methods) consistently for all devices and we measure charge noise at the flank of each Coulomb peak within the *V*_*P*_ range defined by the first peak observable in transport and the last one before onset of a background channel (Supplementary Figs. 2–4). For example, in Fig. 2b we consider Coulomb peaks within the *V*_*P*_ range from 260 mV to 370 mV. The data collected in this systematic way is taken as a basis for comparison between the three different heterostructures in this study.

For each charge noise measurement at a given *V*_*P*_ we acquire 60 s (heterostructure A) or 600 s (heterostructures B, C) long traces of *I*_*SD*_ and split them into 10 (heterostructure A) or 15 windows (heterostructures B, C). We obtain the current noise spectrum *S*_*I*_ by averaging over the 10 (15) windows the discrete Fourier transform of the segments (Methods). We convert *S*_*I*_ to a charge noise spectrum *S*_*ϵ*_ using, for each measurement at a given *V*_*P*_, the unique lever arm from the corresponding Coulomb diamonds and slope of the Coulomb peak to take into account a possible deformation of the charge sensor with the increasing electron number (inset Fig. 2b, Methods, and Supplementary Fig. 5). A representative charge noise spectrum *S*_*ϵ*_ measured at *V*_*P*_ = 360.3 mV is shown in Fig. 2c. We observe an approximate 1/*f* trend at low frequency, pointing towards an ensemble of TLF with a broad range of activation energies affecting charge noise around the charge sensor^41,42^. Figure 2e shows the charge noise *S*_*ϵ*_ at 1 Hz as a function of *V*_*P*_. The charge noise decreases, with a linear trend, with increasing *V*_*P*_, suggesting that, similar to scattering in 2D, screening by an increased electron density shields the electronically active region from noise arising from the heterostructure and the gate stack^43^. From this measurement we extract, for a given device, the minimum measured charge noise at 1 Hz (*S*_*ϵ*,*min*_ circled data point in Fig. 2e) upon variation of *V*_*P*_ in our experimental range. We use *S*_*ϵ*,*min*_ as an informative metric to compare charge noise levels from device to device in a given heterostructure. For a given device, all charge noise spectra *S*_*ϵ*_ are plotted in 3D as a function of *f* and *V*_*P*_ (Fig. 2d). To quantify our observations, we fit the data to the plane $\log {S}_{\epsilon }=-\alpha \log f+\beta {V}_{P}+\gamma$ (Supplementary Note 4). Coefficient *α* = 0.84 ± 0.01 indicates the spectrum power law exponent and coefficient *β* = − 15.6 ± 0.1  mV^−1^ quantifies the change in noise spectrum with increasing plunger gate and, consequently, the susceptibility of charge noise to the increasing electron number in the sensor.

### Distribution of transport properties and charge noise

**Fig. 3:**
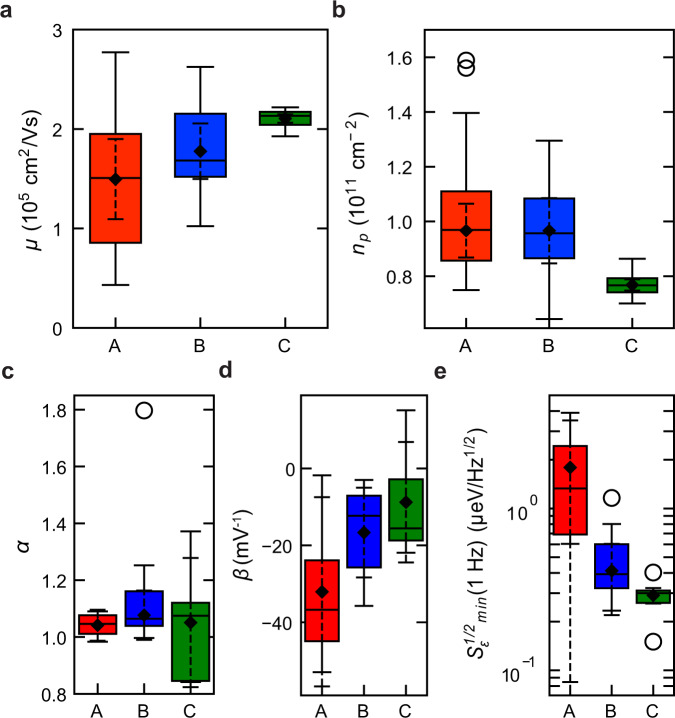
Distribution of transport properties and charge noise. **a**, **b** Distributions of mobility *μ* measured at *n* = 6 × 10^11^ cm^−2^ and percolation density *n*_*p*_ for heterostructure A (red, 20 H-FETs measured, of which 16 reported in ref. ^32^), B (blue, 16 H-FETs measured of which 14 reported in ref. ^32^), and C (green, 22 H-FETs measured). **c**–**e** Distributions of noise spectrum power law exponent *α*, coefficient *β* indicating the change in noise spectrum with increasing *V*_*P*_, and minimum charge noise ${S}_{\epsilon,min}^{1/2}$ within the range of *V*_*P*_ investigated for heterostructure A (red, 4 devices measured), B (blue, 7 devices measured), and C (green, 5 devices measured). Quartile box plots, mode (horizontal line), means (diamonds), 99% confidence intervals of the mean (dashed whiskers), and outliers (circles) are shown.

We have introduced key metrics for 2D electrical transport (*μ*, *n*_*p*_) and charge noise (*α*, *β* and *S*_*ϵ*,*min*_) from Hall bar and quantum dot measurements, respectively. In Fig. 3a–e we compare the distributions of all these metrics for the three heterostructures A, B, C. Each box-plot is obtained from the analysis of measurements in Figs. 1d, e, and 2d repeated on multiple H-FETs or quantum dots, on dies randomly selected from different locations across the 100 mm wafers (Methods). To facilitate a comparison with previous studies, the minimum charge noise at 1 Hz is plotted in Fig. 3e as ${S}_{\epsilon,min}^{1/2}$ and therefore in units of μeV/Hz^½^.

As reported earlier in ref. ^32^, the improvement in both mean values and spread for *μ* and *n*_*p*_ was associated with a reduction of remote impurities when replacing the epitaxial Si cap in heterostructure A with a Si-rich passivation layer in heterostructure B. Moving to heterostructure C, we measure a high mean mobility of (2.10 ± 0.08) × 10^5^ cm^2^/Vs and a low mean percolation density of (7.68 ± 0.37) × 10^10^ cm^−2^, representing an improvement by a factor ≃1.4 and ≃1.3, respectively (compared to heterostructure A). Most strikingly, the 99% confidence intervals of the mean for *μ* and *n*_*p*_ are drastically reduced by a factor ≃9.8 and ≃4.8, respectively. We speculate that these improvements in heterostructure C are associated with the suppression of misfit dislocations at the quantum well/buffer interface, thereby reducing short range scattering and increasing uniformity on a wafer-scale. This interpretation is supported by previous studies of mobility limiting mechanisms as a function of the quantum well thickness in strained Si/SiGe heterostructures^39^. We speculate that further reducing the quantum well thickness could increase surface roughness scattering from the bottom interface, and therefore disorder. Instead, fine-tuning the quantum well thickness between 5 nm and 9 nm might minimize surface roughness scattering whilst still avoiding the formation of misfit dislocations.

We now shift our attention to the results of charge noise measurements. First, the power law exponent *α* (Fig. 3c) shows a mean value ≃1, however the 99% confidence interval and interquartile range increase when moving from heterostructure A to B and C. Next, we observe a decreasing trend for the absolute mean value of coefficient *β* (Fig. 3d), meaning that the noise spectrum is less susceptible to changes in *V*_*P*_. Finally, Fig. 3e shows the distributions for ${S}_{\epsilon,min}^{1/2}$, the minimum charge noise at 1 Hz upon varying *V*_*P*_. We find in heterostructure C an almost order of magnitude reduction in mean ${S}_{\epsilon,min}^{1/2}$ to 0.29 ± 0.02 μeV/Hz^½^. This trend is confirmed by plotting the distributions of maximum charge noise at 1 Hz upon varying *V*_*P*_ (Supplementary Fig. 4). Furthermore, within the distribution of ${S}_{\epsilon,min}^{1/2}$ for heterostructure C, the minimum value of the measured charge noise as a function of *V*_*P*_ and across quantum dots is 0.15 μeV/Hz^½^. These charge noise values are on par or compare favorably to the best values reported previously at 1 Hz in gate defined quantum dots. In multi-electron quantum dots, charge noise of 0.47 μeV/Hz^½^ was reported for Si/SiGe^44^, 0.6 μeV/Hz^½^ (average value, with a minimum of ≤0.2 μeV/Hz^½^) for Ge/SiGe^45^, 0.49 ± 0.1 μeV/Hz^½^ for Si/SiO_2_^46^, and 1 μeV/Hz^½^ for InSb^47^. In single-electron quantum dots, charge noise of 0.33 μeV/Hz^½^ was reported for Si/SiGe^48^ and 7.5 μeV/Hz^½^ for GaAs^49^.

We understand the charge noise trends in Fig. 3c–e by relating them to the evolution of the disorder landscape moving from heterostructures A to B and C, as inferred by the electrical transport measurements in Fig. 3a, b. The narrow distribution of *α* in heterostructure A points to charge noise being dominated from many TLFs possibly located at the low quality semiconductor/dielectric interface and above, albeit other sources of charge noise in the surrounding environment of the quantum dot may be present, such as highly localized misfit dislocations arising from partial strain relaxation in the quantum well or other nearby fluctuators. With a better semiconductor/dielectric interface, the effect of these other nearby fluctuators emerges in heterostructure B and C as a larger spread of the frequency exponent *α*, indicating a nonuniform distribution of activation energies according to the Dutta-Horn model^50^. Yet, the noise spectra still follow a 1/*f*-like behavior (Supplementary Fig. 3), suggesting that TLFs also experience slow temperature fluctuations^42^. The electrical transport measurements support this interpretation: scattering from many remote impurities is dominant in heterostructure A, whereas with a better semiconductor/dielectric interface remote scattering has less impact in the transport metrics of heterostructures B and C.

The decreasing trend in ∣*β*∣ is in line with the observation from electrical transport. As the impurity density decreases from heterostructure A to B and C, charge noise is less affected by an increasing *V*_*P*_, since screening of electrical noise through adding electrons to the charge sensor becomes less effective. While we are not able to measure directly the electron number in the charge sensor, we deem unlikely the hypothesis that charge sensors in heterostructure A are operated with considerably fewer electrons than in heterostructure C. This is because all operation gate voltages in heterostructure A are consistently larger than in heterostructure C (Supplementary Fig. 4), due to the higher disorder.

Finally, the drastic reduction in mean value and spread of ${S}_{\epsilon,min}^{1/2}$ mirrors the evolution of mean value and spread of *n*_*p*_ and *μ*. From heterostructure A to B, a reduction in scattering from remote impurities is likely to result in less charge noise from long-range TLFs. From heterostructure B to C, the reduction in the possible number of dislocations at the quantum well/buffer interface, further reduces the charge noise picked up by quantum dots. This explanation is based on earlier studies of charge noise in strained Si-MOSFETs^27–29^, which showed a correlation between low-frequency noise spectral density and static device parameters. Dislocations at the bottom of the strained channel may act as scattering centers that degrade mobility and as traps for the capture and release of carriers, which causes noise similarly to traps at the dielectric interface.

### Calculated dephasing time and infidelity

To emphasize the improvement of the electrical environment in the semiconductor host, we calculate the dephasing time ${T}_{2}^{\star }$ of charge and spin qubits assuming these qubits experience the same fluctuations as our ^28^Si/SiGe quantum dots. The dephasing time of a qubit (in the quasistatic limit and far-off from a sweet spot) is given by^51,52^

1 $${T}_{2}^{\star }=\frac{h}{\sqrt{2}\pi \sigma }$$

with the Planck constant *h* and the standard deviation

2 $${\sigma }^{2}={\left | \frac{\partial {{{{{{{\mathcal{E}}}}}}}}}{\partial \mu }\right | }^{2}\times 2\int\nolimits_{{f}_{{{{{{{{\rm{low}}}}}}}}}}^{{f}_{{{{{{{{\rm{high}}}}}}}}}}\frac{{S}_{\epsilon }^{2}}{{f}^{\alpha }}df.$$

Importantly, both the charge noise amplitude ${S}_{\epsilon }^{2}(f)$ and the noise exponent *α* have a strong impact on the dephasing time while the low and high frequency cut-off, *f*_low_ and *f*_high_, given by the duration of the experiment have a weaker impact. The prefactor $\left|\frac{\partial {{{{{{{\mathcal{E}}}}}}}}}{\partial \mu }\right|$ translates shifts in chemical potential of the charge sensor into energy shifts of the qubit and depends on many parameters such as the type of qubit and the device itself. We find $\left|\frac{\partial {{{{{{{\mathcal{E}}}}}}}}}{\partial \mu }\right |=1$ for a charge qubit^53^ and $\left|\frac{\partial {{{{{{{\mathcal{E}}}}}}}}}{\partial \mu }\right|\approx 1{0}^{-5}$ for an uncoupled spin- qubit^44^ (see Supplementary Note 7 for a derivation of these numbers and the used frequency bandwidths).

**Fig. 4:**
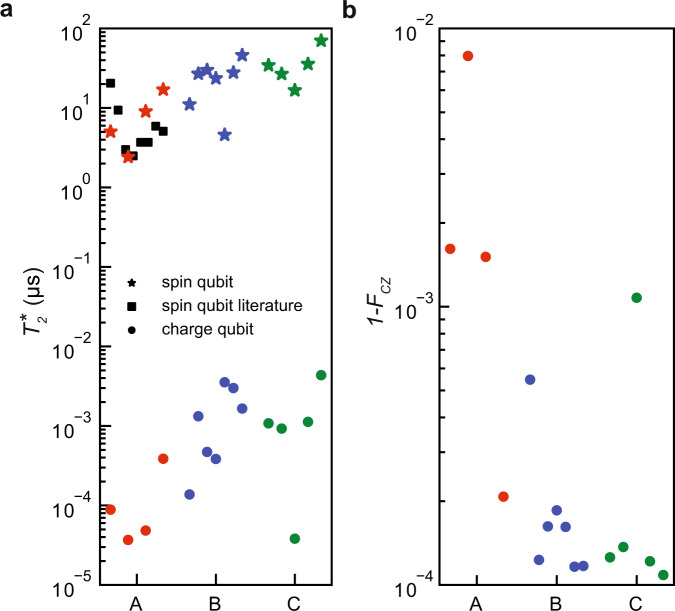
Calculated dephasing times and infidelity. **a** Computed dephasing times ${T}_{2}^{\star }$ of a charge qubit (circle) and of a spin-qubit (star) using *S*_*ϵ*,*min*_ from heterostructure A (red), B (blue), C (green). Eq. (1) was used to compute ${T}_{2}^{\star }$ as a function of *S*_*ϵ*_ and *α* from Fig. 3 with frequency cutoffs (*f*_*min*_, *f*_*max*_) = (1.6 mHz, 33 GHz) and (*f*_*min*_, *f*_*max*_) = (1.6 mHz, 10 kHz). Literature values (squares) are taken from refs. ^6,10^. **b** Simulated infidelity of a CZ-gate between two spin qubits following the ref. ^6^ using *S*_*ϵ*_ and *α* from heterostructure A (red), B (blue), C (green) in Fig. 3 as input for barrier fluctuations.

Figure 4a shows the computed dephasing times of charge qubits (circle) and spin qubits (star) for all three heterostructures. These calculations represent a best case scenario, since we use the distribution of measured *S*_*ϵ*,*min*_ from Fig. 3 as input parameter for each heterostructure. The improvements in our material can be best seen by investigating ${T}_{2}^{\star }$ of the charge qubit since it is directly affected by charge noise. Our theoretical extrapolation shows two orders of magnitude improvement in ${T}_{2}^{\star }$ by switching from heterostructures A to heterostructures B and C. One order is gained from the reduced charge noise amplitude and another order is gained through a more beneficial noise exponent *α* > 1. Note, that the integration regimes differ for spin and charge qubits due to the different experimental setups and operation speeds^44,53^. For potential spin qubits in heterostructure A the calculated ${T}_{2}^{\star }$ shows an average ${\overline{T}}_{2}^{\star }=8.4\pm 5.6$ μs. This distribution compares well with the distribution ${\overline{T}}_{2}^{\star }=6.7\pm 5.6$ μs of experimental ${T}_{2}^{\star }$ data from state-of-the-art semiconductor spin qubits in materials with similar stacks as in heterostructure A^6,10^. Note that while such comparisons oversimplify actual semiconductor spin-qubit devices by reducing them to a single number, they fulfill two aims. They allow us to benchmark the computed performance of heterostructure A to past experiments and provide a prognosis on the qubit quality in novel material stacks. Heterostructures B and C, in this case, may support average dephasing times of ${\overline{T}}_{2}^{\star }=24.3\pm 12.5$ μs and ${\overline{T}}_{2}^{\star }=36.7\pm 18$ μs, respectively. The highest values ${T}_{2}^{\star }=70.1$ μs hints towards a possible long dephasing time for spin qubits, previously only reported in ref. ^2^.

Figure 4b shows the simulated infidelity, a metric to measure the closeness to the ideal operation, of a universal CZ-gate between two spin qubits following ref. ^6^ and Supplementary Note 7. Note that the device used in ref. ^6^ has the same architecture as our test devices. In the CZ-gate simulation, noise couples in dominantly via barrier voltage fluctuations which affects the interaction between the electron spins. Again, we use the charge noise amplitude *S*_*ϵ*,*min*_ and exponent *α* from the quantum dot experiments in Fig. 3 as input for the simulations. The simulations show an averaged average gate infidelity $1-{\overline{F}}_{{\scriptstyle{{{{{\rm{CZ}}}}}}}}=0.02\pm 0.01\%$ which means on average a single error every 5000 runs. We also observe a saturation value close to 1 − *F* = 10^−4^ which arises from single-qubit dephasing ${T}_{2}^{\star }=20$ μs used in the simulations estimated from nuclear spin noise due to a 800 ppm concentration of the ^29^Si silicon isotope which has a non-zero nuclear spin^44^.

## Discussion

In summary, we have measured electron transport and charge noise in ^28^Si/SiGe heterostructures where we improve the semiconductor/dielectric interface, by adopting an amorphous Si-rich passivation, and the structural quality of the quantum well, by reducing the quantum well thickness significantly below the Matthew-Blakeslee critical thickness for strain relaxation. We relate disorder in 2D to charge noise in quantum dots by following a statistical approach to measurements. A reduction of remote impurities and dislocations nearby the quantum well is connected with the key improvements in the scattering properties of the 2D electron gas, such as mobility and percolation density, and their uniformity across a 100 mm wafer. The trend observed from electron transport in 2D is compatible with the observations from measurements of charge noise in quantum dots. As remote impurities are reduced, charge noise becomes more sensitive to local fluctuators nearby the quantum well and less subject to screening by an increased number of electrons in the dot. Furthermore, with this materials optimization, we achieve a statistical improvement of nearly one order of magnitude in the charge noise supported by quantum dots. Using the charge noise distribution as input parameter and benchmarking against published spin-qubit data, we predict that our optimized semiconductor host could support long-lived and high-fidelity spin qubits. We envisage that further materials improvements in the structural quality of the quantum well, in addition to the commonly considered semiconductor/dielectric interface, may lead systematically to quantum dots with less noise and to better qubit performance.

## Methods

### Si/SiGe heterostructure growth

The ^28^Si/SiGe heterostructures are grown on a 100-mm n-type Si(001) substrate using an Epsilon 2000 (ASMI) reduced pressure chemical vapor deposition reactor. The reactor is equipped with a ^28^SiH_4_ gas cylinder (1% dilution in H_2_) for the growth of isotopically enriched ^28^Si. The ^28^SiH_4_ gas was obtained by reducing ^28^SiF_4_ with a residual ^29^Si concentration of 0.08%^54^. Starting from the Si substrate, the layer sequence of all heterostructures comprises a 3 μm step-graded Si_(1−*x*)_Ge_*x*_ layer with a final Ge concentration of *x* = 0.3 achieved in four grading steps (*x* = 0.07, 0.14, 0.21, and 0.3), followed by a 2.4 μm Si_0.7_Ge_0.3_ strain-relaxed buffer. The heterostructures differ for the active layers on top of the strain-relaxed buffer. Heterostructure A has a 9 nm tensile strained ^28^Si quantum well, a 30 nm Si_0.7_Ge_0.3_ barrier, and a sacrificial 1 nm epitaxial Si cap. Heterostructure B has an 9 nm tensile strained ^28^Si quantum well, a 30 nm Si_0.7_Ge_0.3_ barrier, and a sacrificial passivated Si cap grown at 500 °C. Heterostructure C has a 5 nm tensile strained ^28^Si quantum well, a 30 nm Si_0.7_Ge_0.3_ barrier, and a sacrificial passivated Si cap grown at 500 °C. A typical secondary ions mass spectrometry of our heterostructures is reported in Supplementary Fig. S13 of ref. ^40^ and the Ge concentration in the SiGe layers is confirmed by quantitative electron energy loss spectroscopy (EELS).

### Device fabrication

The fabrication process for Hall-bar shaped heterostructure field effect transistors (H-FETs) involves: reactive ion etching of mesa-trench to isolate the two-dimensional electron gas; P-ion implantation and activation by rapid thermal annealing at 700 °C; atomic layer deposition of a 10-nm-thick Al_2_O_3_ gate oxide; deposition of thick dielectric pads to protect gate oxide during subsequent wire bonding step; sputtering of Al gate; electron beam evaporation of Ti:Pt to create ohmic contacts to the two-dimensional electron gas via doped areas. All patterning is done by optical lithography. Double quantum dot devices are fabricated on wafer coupons from the same H-FET fabrication run and share the process steps listed above. Double-quantum dot devices feature a single layer gate metallization and further require electron beam lithography, evaporation of Al (27 nm) or Ti:Pd (3:17 nm) thin film metal gate, lift-off, ALD of a 5 nm thick Al_2_O_3_ insulating layer, and a global top-gate.

### Electrical characterization of H-FETs

Hall-bar H-FETs measurements are performed in an attoDRY2100 variable temperature insert refrigerator at a base temperature of 1.7 K^32^. We apply a source-drain bias of 100 μV and measure the source-drain current *I*_SD_, the longitudinal voltage *V*_*xx*_, and the transverse Hall voltage *V*_*xy*_ as function of the top gate voltage *V*_*g*_ and the external perpendicular magnetic field *B*. From here we calculate the longitudinal resistivity *ρ*_*xx*_ and transverse Hall resistivity *ρ*_*xy*_. The Hall electron density *n* is obtained from the linear relationship *ρ*_*xy*_ = *B*/*en* at low magnetic fields. The carrier mobility *μ* is extracted from the relationship *σ*_*xx*_ = *neμ*, where *e* is the electron charge. The percolation density *n*_*p*_ is extracted by fitting the longitudinal conductivity *σ*_*xx*_ to the relation ${\sigma }_{xx}\propto {(n-{n}_{p})}^{1.31}$. Here *σ*_*xx*_ is obtained via tensor inversion of *ρ*_*xx*_ at *B* = 0. The box plots in Fig. 3a, b for heterostructure A (red) and B (blue) expand previously published data in Fig. 2f, e of ref. ^32^ by considering measurements of 4 additional H-FETs for heterostructure A (20 H-FETs in total) and of 2 additional H-FETs for heterostructure B (16 H-FETs in total).

### Electrical characterization of quantum dots

Measurements of the multi-electron quantum dots defined in the charge sensor are performed in a Leiden cryogenic dilution refrigerator with a mixing chamber base temperature *T*_MC_ = 50 mK^40^. The devices are tuned systematically with the following procedure. We sweep all gate voltages (*V*_*SDRAcc*_, *V*_*SDRB*_, *V*_*P*_, *V*_*SDLB*_, and *V*_*SDLAcc*_) from 0 V towards more positive bias, until a source-drain current *I*_*SD*_ of ≈1 nA is measured, indicating that a conductive channel has formed in the device. We then reduce the barrier voltages to find the pinch-off voltages for each barrier. Subsequently, we measure *I*_*SD*_ as a function of *V*_*SDLB*_ and *V*_*SDRB*_ and from this 2D map we find a set of gate voltage parameters so that Coulomb blockade peaks are visible. We then fix the barrier voltages and sweep V_*P*_ to count how many clearly defined Coulomb peaks are observed before onset of a background current. The quantum dot is tuned to show at least 9 Coulomb peaks, so that noise spectra may be fitted as in Fig. 2d with meaningful error bars. If we see less than 9 Coulomb peaks we readjust the accumulation gate voltages *V*_*SDRAcc*_, and *V*_*SDLAcc*_, and repeat the 2D scan of *V*_*SDLB*_ against *V*_*SDRB*_. In one case (device 2 of heterostructure A), we tuned device to show past 5 Coulomb peaks and still performed the fit of the charge noise spectra similar to the one shown in Fig. 2d. Further details on the extraction of the lever arms and operation gate voltages of the devices are provided in Supplementary Figs 4 and 5. We estimate an electron temperature of 190 mK by fitting Coulomb blockade peaks (see Supplementary Fig. 2 in ref. ^32^) measured on quantum dot devices.

For heterostructure A we apply a source drain bias of 100 μV (1 device) or 150 μV (3 devices) across the quantum dot, finite gate voltages across the operation gates of the dot, and finite gate voltages across the screening gates. We measure the current *I*_*SD*_ and the current noise spectrum *S*_*I*_ on the left side of the Coulomb peak where ∣*dI*/*dV*_*P*_∣ is largest. We use a sampling rate of 1 kHz for 1 min using a Keithley DMM6500 multimeter. The spectra are then divided into 10 segments of equal length and we use a Fourier transform to convert from time-domain to frequency-domain for a frequency range of 167 mHz–500 Hz. We set the upper limit of the frequency spectra at 10 Hz, to avoid influences from a broad peak at around 150 Hz coming from the setup (Supplementary Fig. 3). A peak in the power spectral density at 9 Hz is removed from the analysis since it is an artifact of the pre-amplifier. To convert the current noise spectrum to a charge noise spectrum, we use the formula^20^

3 $${S}_{\epsilon }=\frac{{a}^{2}{S}_{I}}{|dI/d{V}_{P}{|}^{2}}$$

where *a* is the lever arm and ∣*dI*/*dV*_*P*_∣ is the slope of the Coulomb peak at the plunger voltage used to acquire the time trace.

The charge noise measurements conditions have been slightly modified from sample A to sample B, C to extend the probed frequency range from 100 μHz to 10 μHz. For heterostructures B and C we apply a source drain bias of 150 μV across the quantum dot, finite gate voltages across the operation gates of the quantum dot, and we apply 0 V to all other gates. We measure the current *I*_*SD*_ and the current noise spectrum *S*_*I*_ on the left side of the Coulomb peak where ∣*dI*/*dV*_*P*_∣ is largest. We use a sampling rate of 1 kHz for 10 min using a Keithley DMM6500 multimeter. The spectra are then divided into 15 segments of equal length and we use a Fourier transform to convert from time-domain to frequency-domain for a frequency range of 25 mHz–500 Hz. We set the upper limit of the frequency spectra at 10 Hz, to avoid influences from a broad peak at around 150 Hz coming from the setup. We use Eq. (3) to convert the current noise spectrum to a charge noise spectrum.

### (Scanning) Transmission Electron Microscopy

For structural characterization with (S)TEM, we prepared cross-sections of the quantum well heterostructures by using a Focused Ion Beam (Helios 600 dual beam microscope). Atomically resolved HAADF STEM data was acquired in a probe corrected TITAN microscope operated at 300 kV. Quantitative EELS was carried out in a TECNAI F20 microscope operated at 200 kV with approximately 2 eV energy resolution and 1 eV energy dispersion. Principal Component Analysis (PCA) was applied to the spectrum images to enhance S/N ratio.

## Supplementary information

Supplementary Information

Peer Review File

## Data Availability

All data included in this work are available from the 4TU.ResearchData international data repository at 10.4121/20418579.

## References

[1] Vandersypen LMK, Eriksson MA (2019). Quantum computing with semiconductor spins. Phys. Today.

[2] Veldhorst M (2015). A two-qubit logic gate in silicon. Nature.

[3] Stano, P. & Loss, D. Review of performance metrics of spin qubits in gated semiconducting nanostructures. *Nat. Rev. Phys.* **4**, 672–688 (2022).

[4] Zwerver AMJ (2022). Qubits made by advanced semiconductor manufacturing. Nat. Electronics.

[5] Watson TF (2018). A programmable two-qubit quantum processor in silicon. Nature.

[6] Xue X (2022). Quantum logic with spin qubits crossing the surface code threshold. Nature.

[7] Noiri A (2022). Fast universal quantum gate above the fault-tolerance threshold in silicon. Nature.

[8] Ma̧dzik MT (2022). Precision tomography of a three-qubit donor quantum processor in silicon. Nature.

[9] Mills AR (2022). Two-qubit silicon quantum processor with operation fidelity exceeding 99%. Sci. Adv..

[10] Philips SGJ (2022). Universal control of a six-qubit quantum processor in silicon. Nature.

[11] Samkharadze N (2018). Strong spin-photon coupling in silicon. Science.

[12] Zajac DM (2018). Resonantly driven CNOT gate for electron spins. Science.

[13] Borjans F, Croot XG, Mi X, Gullans MJ, Petta JR (2020). Resonant microwave-mediated interactions between distant electron spins. Nature.

[14] Harvey-Collard P (2022). Coherent spin-spin coupling mediated by virtual microwave photons. Phys. Rev. X.

[15] Yoneda J (2021). Coherent spin qubit transport in silicon. Nat. Commun..

[16] Noiri A (2022). A shuttling-based two-qubit logic gate for linking distant silicon quantum processors. Nat. Commun..

[17] Vandersypen LMK (2017). Interfacing spin qubits in quantum dots and donors-hot, dense, and coherent. npj Quantum Inf..

[18] Yoneda J (2018). A quantum-dot spin qubit with coherence limited by charge noise and fidelity higher than 99.9%. Nat. Nanotechnol..

[19] Paladino E, Galperin YM, Falci G, Altshuler BL (2014). 1/f noise: Implications for solid-state quantum information. Rev. Modern Phys..

[20] Connors EJ, Nelson JJ, Qiao H, Edge LF, Nichol JM (2019). Low-frequency charge noise in Si/SiGe quantum dots. Phys. Rev. B.

[21] Connors EJ, Nelson J, Edge LF, Nichol JM (2022). Charge-noise spectroscopy of Si/SiGe quantum dots via dynamically-decoupled exchange oscillations. Nat. Commun..

[22] Culcer D, Hu X, Das Sarma S (2009). Dephasing of Si spin qubits due to charge noise. Appl. Phys. Lett..

[23] Dekker C (1991). Spontaneous resistance switching and low-frequency noise in quantum point contacts. Phys. Rev. Lett..

[24] Sakamoto T, Nakamura Y, Nakamura K (1998). Distributions of single-carrier traps in GaAs/Al_*x*_Ga_1−*x*_As heterostructures. Appl. Phys. Lett..

[25] Liefrink F, Dijkhuis JI, Houten HV (1994). Low-frequency noise in quantum point contacts. Semiconductor Sci. Technol..

[26] Ramon G, Hu X (2010). Decoherence of spin qubits due to a nearby charge fluctuator in gate-defined double dots. Phys. Rev. B.

[27] Hua W-C, Lee MH, Chen PS, Tsai M-J, Liu CW (2005). Threading dislocation induced low frequency noise in strained-Si nMOSFETs. IEEE Electron Device Letters.

[28] Lee, M. H. et al. Comprehensive low-frequency and RF noise characteristics in strained-Si NMOSFETs. In: IEEE International Electron Devices Meeting 2003, pp. 3–61364 (2003).

[29] Simoen E (2006). Processing aspects in the low-frequency noise of nMOSFETs on strained-silicon substrates. IEEE Trans. Electron Dev..

[30] Monroe D (1993). Comparison of mobility-limiting mechanisms in high-mobility Si_1−*x*_Ge_*x*_ heterostructures. J. Vac. Sci. Technol. B.

[31] Xue X (2021). Cmos-based cryogenic control of silicon quantum circuits. Nature.

[32] Degli Esposti D (2022). Wafer-scale low-disorder 2DEG in ^28^Si/SiGe without an epitaxial Si cap. Appl. Phys. Lett..

[33] Matthews JW, Blakeslee AE (1974). Defects in epitaxial multilayers: I. Misfit dislocations. J. Crystal Growth.

[34] People R, Bean JC (1985). Calculation of critical layer thickness versus lattice mismatch for Ge_*x*_Si_1−*x*_/Si strained-layer heterostructures. Appl. Phys. Lett..

[35] Ismail K (1996). Effect of dislocations in strained Si/SiGe on electron mobility. J. Vac. Sci. Technol. B.

[36] Liu Y (2022). Role of critical thickness in SiGe/Si/SiGe heterostructure design for qubits. J. Appl. Phys..

[37] Paquelet Wuetz B (2020). Multiplexed quantum transport using commercial off-the-shelf CMOS at sub-kelvin temperatures. npj Quantum Inf..

[38] Tracy LA (2009). Observation of percolation-induced two-dimensional metal-insulator transition in a Si MOSFET. Phys. Rev. B.

[39] Ismail K (1994). Identification of a mobility-limiting scattering mechanism in modulation-doped Si/SiGe heterostructures. Phys. Rev. Lett..

[40] Paquelet Wuetz B (2022). Atomic fluctuations lifting the energy degeneracy in Si/SiGe quantum dots. Nat. Commun..

[41] Kogan, S. *Electronic noise and fluctuations in solids* (Cambridge University Press, 1996).

[42] Ahn S, Sarma SD, Kestner JP (2021). Microscopic bath effects on noise spectra in semiconductor quantum dot qubits. Phys. Rev. B.

[43] Thorgrimsson B (2017). Extending the coherence of a quantum dot hybrid qubit. npj Quantum Inf..

[44] Struck T (2020). Low-frequency spin qubit energy splitting noise in highly purified ^28^Si/SiGe. npj Quantum Inf..

[45] Lodari M (2021). Low percolation density and charge noise with holes in germanium. Mater. Quantum Technol..

[46] Freeman BM, Schoenfield JS, Jiang H (2016). Comparison of low frequency charge noise in identically patterned Si/SiO_2_ and Si/SiGe quantum dots. Appl. Phys. Lett..

[47] Jekat F (2020). Exfoliated hexagonal BN as gate dielectric for InSb nanowire quantum dots with improved gate hysteresis and charge noise. Appl. Phys. Lett..

[48] Mi X, Kohler S, Petta JR (2018). Landau-Zener interferometry of valley-orbit states in Si/SiGe double quantum dots. Phys. Rev. B.

[49] Basset J (2014). Evaluating charge noise acting on semiconductor quantum dots in the circuit quantum electrodynamics architecture. Appl. Phys. Lett..

[50] Dutta P, Dimon P, Horn PM (1979). Energy scales for noise processes in metals. Phys. Rev. Lett..

[51] Shnirman A, Makhlin Y, Schön G (2002). Noise and decoherence in quantum two-level systems. Phys. Scripta.

[52] Bermeister A, Keith D, Culcer D (2014). Charge noise, spin-orbit coupling, and dephasing of single-spin qubits. Appl. Phys. Lett..

[53] MacQuarrie ER (2020). Progress toward a capacitively mediated CNOT between two charge qubits in Si/SiGe. npj Quantum Inf..

[54] Sabbagh D (2019). Quantum Transport Properties of Industrial ^28^Si/^28^SiO_2_. Phys. Rev. Appl..

