# Author Correction: Reducing charge noise in quantum dots by using thin silicon quantum wells

**DOI:** 10.1038/s41467-023-37548-z

**Published:** 2023-04-06

**Authors:** Brian Paquelet Wuetz, Davide Degli Esposti, Anne-Marije J. Zwerver, Sergey V. Amitonov, Marc Botifoll, Jordi Arbiol, Amir Sammak, Lieven M. K. Vandersypen, Maximilian Russ, Giordano Scappucci

**Affiliations:** 1 QuTech and Kavli Institute of Nanoscience, Delft University of Technology, PO Box 5046, 2600 GA Delft, The Netherlands; 2 QuTech and Netherlands Organisation for Applied Scientific Research (TNO), Delft, The Netherlands; 3 Catalan Institute of Nanoscience and Nanotechnology (ICN2), CSIC and BIST, Campus UAB, Bellaterra, 08193 Barcelona, Catalonia Spain; 4 ICREA, Pg. Lluís Companys 23, 08020 Barcelona, Catalonia Spain

**Keywords:** Quantum dots, Spintronics

Correction to: *Nature Communications* 10.1038/s41467-023-36951-w, published online 13 March 2023

The original version of this Article omitted from the author list the author Amir Sammak who is from the ‘QuTech and Netherlands Organisation for Applied Scientific Research (TNO), Delft, The Netherlands’. This has been corrected in both the PDF and HTML versions of the Article.

