# Supplementary material for: Reducing charge noise in quantum dots by using thin silicon quantum wells
Source: Nat Commun. 2023 Mar 13;14:1385. doi: 10.1038/s41467-023-36951-w (PMC10011559; doi:10.1038/s41467-023-36951-w)
Supplement: Supplementary file 1 — Supplementary Information [file 41467_2023_36951_MOESM1_ESM.pdf]

Supplementary Information: Reducing charge noise in quantum dots by using thin silicon quantum wells

B. Paquelet Wuetz,[1] D. Degli Esposti,[1] A.M.J. Zwerver,[1] S.V. Amitonov,[1,2] M. Botifoll,[3] J. Arbiol,[3,4] A. Sammak,[2] L.M.K. Vandersypen,[1] M. Russ,[1] and G. Scappucci[1,*]

[1] *QuTech and Kavli Institute of Nanoscience, Delft University of Technology, PO Box 5046, 2600 GA Delft, The Netherlands*
[2] *QuTech and Netherlands Organisation for Applied Scientific Research (TNO), Stieltjesweg 1, 2628 CK Delft, The Netherlands*
[3] *Catalan Institute of Nanoscience and Nanotechnology (ICN2), CSIC and BIST, Campus UAB, Bellaterra, 08193 Barcelona, Catalonia, Spain*
[4] *ICREA, Pg. Lluís Companys 23, 08010 Barcelona, Catalonia, Spain*

(Dated: February 24, 2023)

CONTENTS

1. Measurement of the thickness of the quantum wells 2

2. Charge noise measurements 4

3. Noise spectra for a quantum dot from heterostructure C 5

4. Frequency and gate voltage dependence of noise spectra 6

5. Operation gate voltages for charge sensor quantum dots 7

6. Lever arm extraction 8

7. Simulations of dephasing times and gate fidelities 8
   Dephasing of charge qubit 9
   Dephasing of spin qubit 9
   Gate fidelity simulations 9

References 9

* g.scappucci@tudelft.nl

1. MEASUREMENT OF THE THICKNESS OF THE QUANTUM WELLS

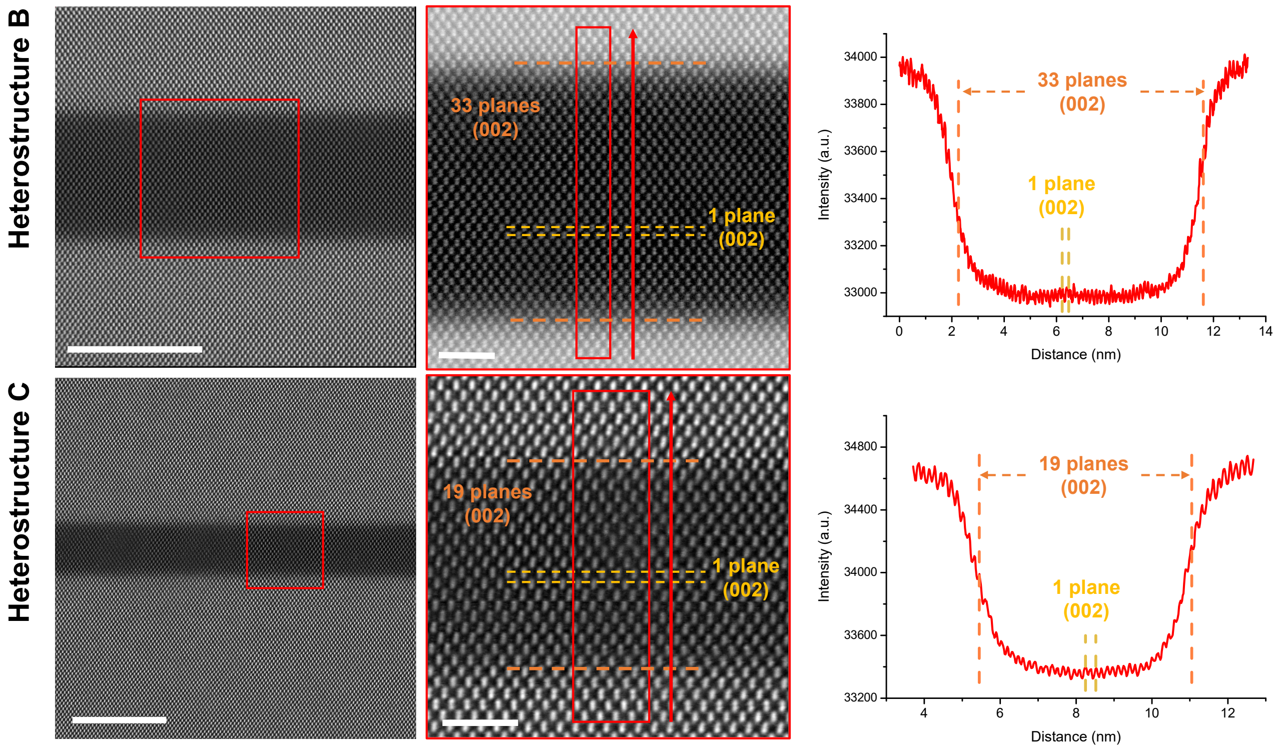

Figure S1. Method for computing the thickness of the quantum well based on the counting of the (002) horizontal planes, which reduces the uncertainty and bias associated to properly detecting the margins of the quantum well, for both heterostructures B and C. Scale bars are 10 nm (images in left column) and 2 nm (images in center column))

To avoid possible errors associated with calibration, we measure the thickness of the Si layer in the quantum wells (t_{qw}) for heterostructures B and C by considering the interplanar spacing of the horizontal planes (002) of the quantum well (d_{qw}) and of the underlying the strain-relaxed SiGe buffer layer (d_{buffer}). For the $Si_{1-x}Ge_x$ buffer layer, we consider the stoichiometry x as measured by means of quantitative EELS and calculate the theoretical expected cell parameter a_{cell} using the following approximation of Vegard's law:

$$a_{cell} = \; a_{Si} + 0.2x + 0.027x^2, \tag{1}$$

where a_{Si} = 5.431 Å is the cell parameter of the diamond cubic Si crystal phase. To calculate d_{buff} we use the formula for the interplanar distance of the desired plane (002) of a diamond cubic system:

$$d_{hkl} = \frac{a_{cell}}{\sqrt{h^2+k^2+l^2}} \; = \; \frac{a_{cell}}{\sqrt{0^2+0^2+2^2}} \; = \; \frac{a_{cell}}{2}. \tag{2}$$

Since the quantum well is strained, d_{qw} is found by considering the average dilatation δ of the quantum well (002) planes with respect the (002) planes of the buffer. The dilatation δ is obtained experimentally by Geometrical Phase Analysis (GPA). The standard deviation of GPA is high for dilatation close to 0, as happens with the (220) epitaxial planes, for which the method is not the preferred choice. Nevertheless, for the larger dilatation of the (002) planes, the relatively smaller standard deviation makes the measurement significative. As a result, d_{qw} is computed by:

$$d_{qw} = \; d_{buff}\left(1+\delta\right). \tag{3}$$

Finally, the thickness of the quantum well is given by:

$$t_{qw} = n_{qw} d_{qw}, \tag{4}$$

where we count the number of planes forming the quantum well (n_{qw}) and multiply by d_{qw}. Therefore, the expected uncertainty of the thickness measurement lies in whether the initial and last plane of the well are being considered or not, *i.e.* the standard deviation is given by $\sigma = 2d_{qw}$.

With this in mind, for heterostructure B, where $x = 0.31$, four different measurements counting the (002) planes were performed in different regions of the quantum well, $n_{qw} = 33$ (3 times) and 34. With an average experimental δ of -1.6±0.2 %, we obtain $d_{qw} = 2.704 \pm 0.007$ Å, resulting in an average thickness $t_{qw} = 9.0 \pm 0.5$ nm.

For heterostructure C, $x = 0.31$ and two measurements counting the (002) planes were performed, $n_{qw} = 19$ and 20. With an average experimental δ of -1.7±0.5 %, we obtain $d_{qw} = 2.701 \pm 0.014$ Å, resulting in an average thickness $t_{qw} = 5.3 \pm 0.5$ nm.

2. CHARGE NOISE MEASUREMENTS

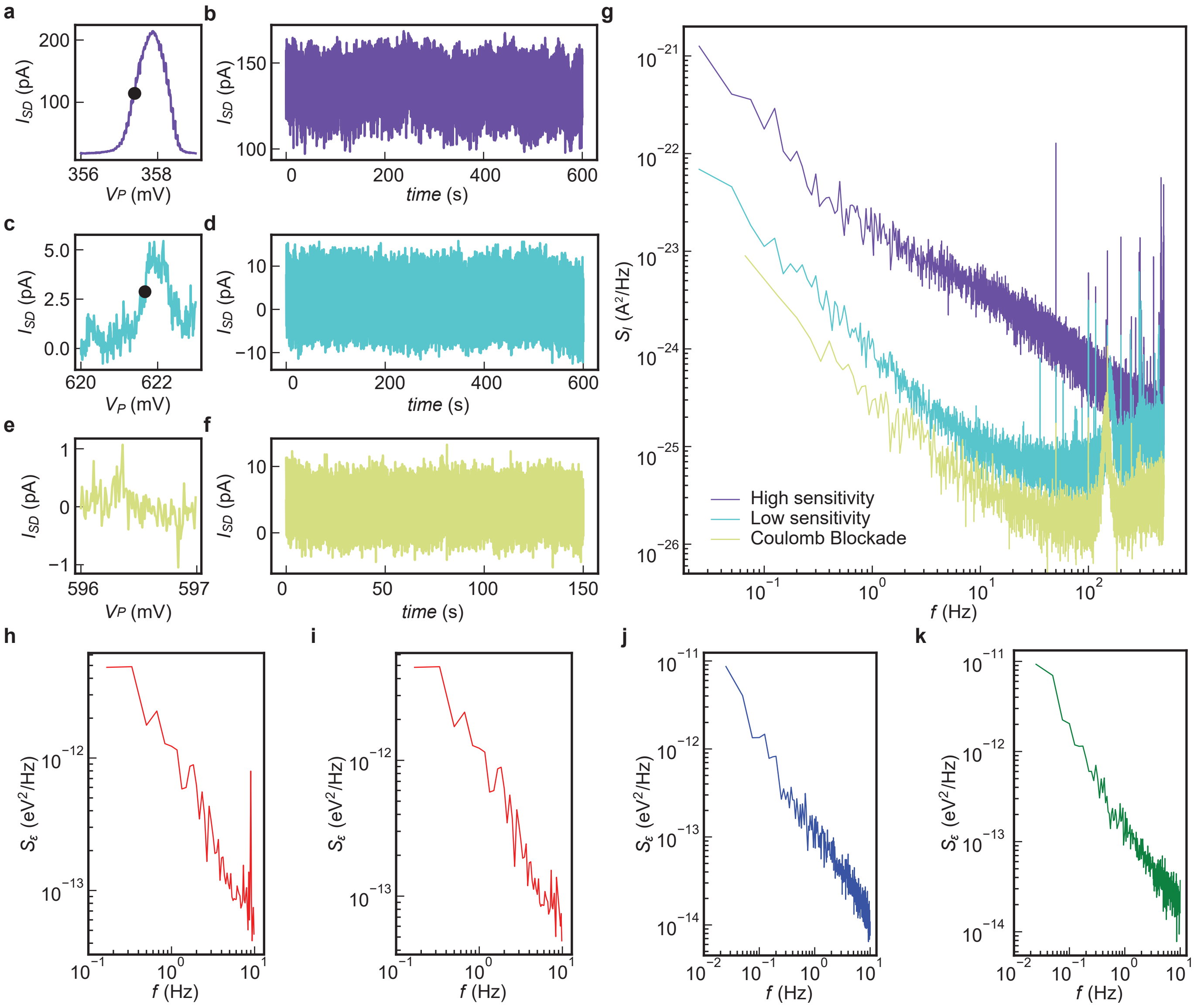

Figure S2. Comparison, for illustration purposes, of charge noise measurements spectra under different conditions. **a** Coulomb peak with large derivative dI_{SD}/dV_P and **b** time-resolved I_{SD} measured at the flank of the Coulomb peak (dot in **a**). Measurements are from a device from heterostructure C. **c** Coulomb peak with smaller derivative dI_{SD}/dV_P and **d** time-resolved I_{SD} measured at the flank of the Coulomb peak (dot in **c**). Measurements are from a device from heterostructure B. **e** Coulomb blockade and **f** time-resolved I_{SD} measured on a test device from heterostructure B, indicative of the noise floor of our measurement setup. The time traces in **b**,**d**,**f** show a consistent decrease in the noise bandwidth going from the most sensitive ($\Delta I_{SD} \simeq 50$ pA in **b**) to the less sensitive ($\Delta I_{SD} \simeq 10$ pA in **f**) configuration. **g** Comparison of the current noise spectrum under different sensitivity conditions. Purple (high sensitivity), cyan (low-sensitivity), and lemon (noise floor) curves shows $S_I(f)$ obtained from measurements in **b**, **d**, and **f**, respectively. Lemon and cyan curves show a broad interference peak at 150 Hz, as well as a flattening out of the curve at ≈40 Hz. **h** Charge noise measurement of heterostructure A with an interference peak at 9 Hz arising from the measurement module. In **i** we remove the interference peak from the analysis. **j**,**k** Charge noise of a device from heterostructure B and C, respectively, measured with a different measurement module compared to **h** showing no interference peak.

3. NOISE SPECTRA FOR A QUANTUM DOT FROM HETEROSTRUCTURE C

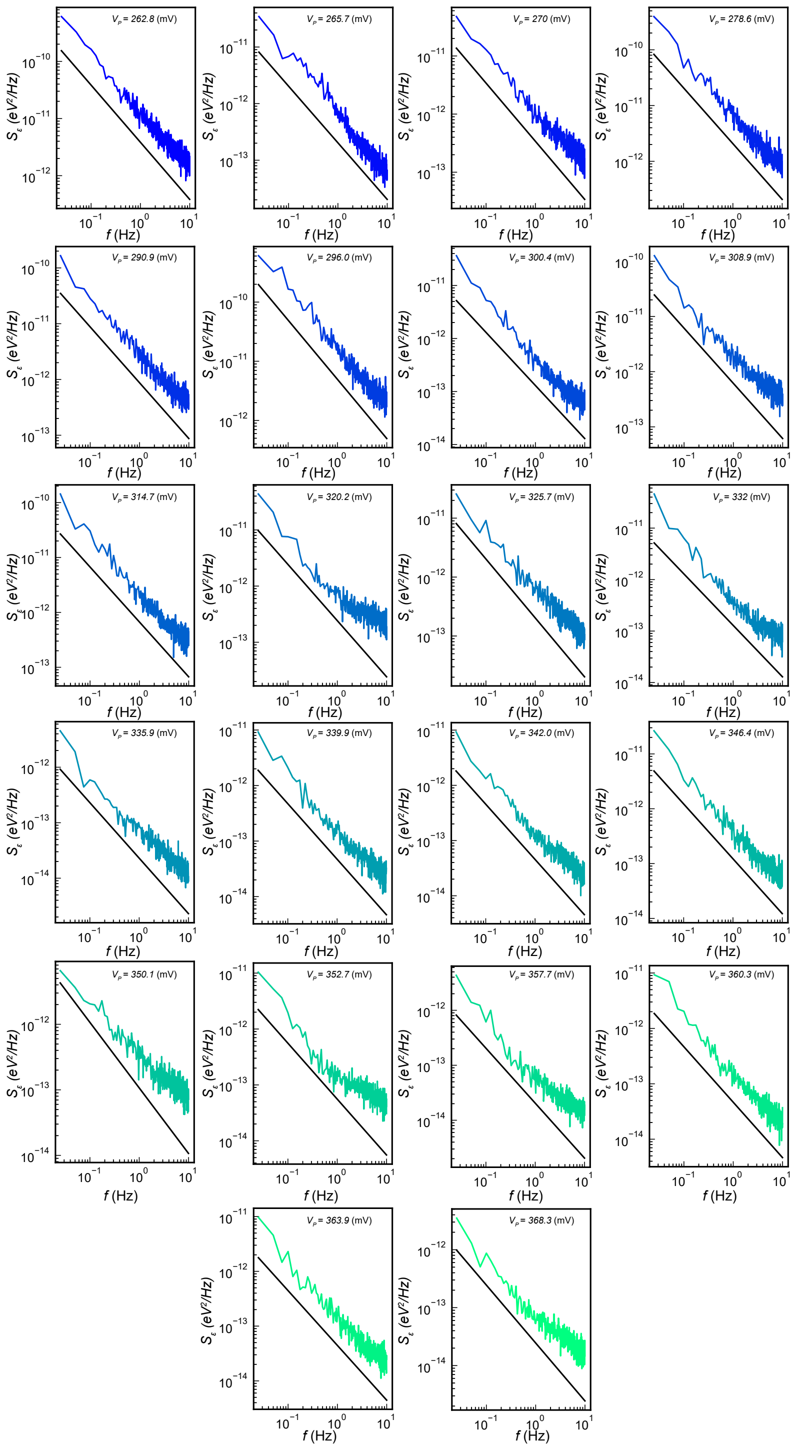

Figure S3. Charge noise spectra $S_\epsilon(f)$ at different plunger gate voltage V_P from a quantum dot from heterostructure C. The same data is plotted in Fig. 2d in three dimensions. The black trendline shows a $1/f$ dependence.

4. FREQUENCY AND GATE VOLTAGE DEPENDENCE OF NOISE SPECTRA

We formulated the equation for the plane in Fig. 2d

$$\log S_\epsilon = -\alpha \log f + \beta V_P + \gamma \tag{5}$$

to capture the experimental dependence of $S_\epsilon(f, V_P)$ observed when S_ϵ and f are plotted on a log scale. Eq. 5 expands the known expression describing the low-frequency dependence of the charge noise spectrum[1, 2]

$$S_\epsilon = \frac{A}{f^\alpha}, \tag{6}$$

where A is a constant prefactor describing the noise strength evaluated at 1 Hz. We now multiply a phenomenological prefactor $e^{\beta V_P}$, that describes the reduction in susceptibility of the quantum dot to electric noise as a function of plunger voltage V_P [3]. This yields the expression

$$S_\epsilon = \frac{A}{f^\alpha} e^{\beta V_P}. \tag{7}$$

By taking the logarithm of Eq. 7 and defining $\gamma = \log(A)$ as the unscreened noise, we arrive at the expression Eq. 5 in the main text.

5. OPERATION GATE VOLTAGES FOR CHARGE SENSOR QUANTUM DOTS

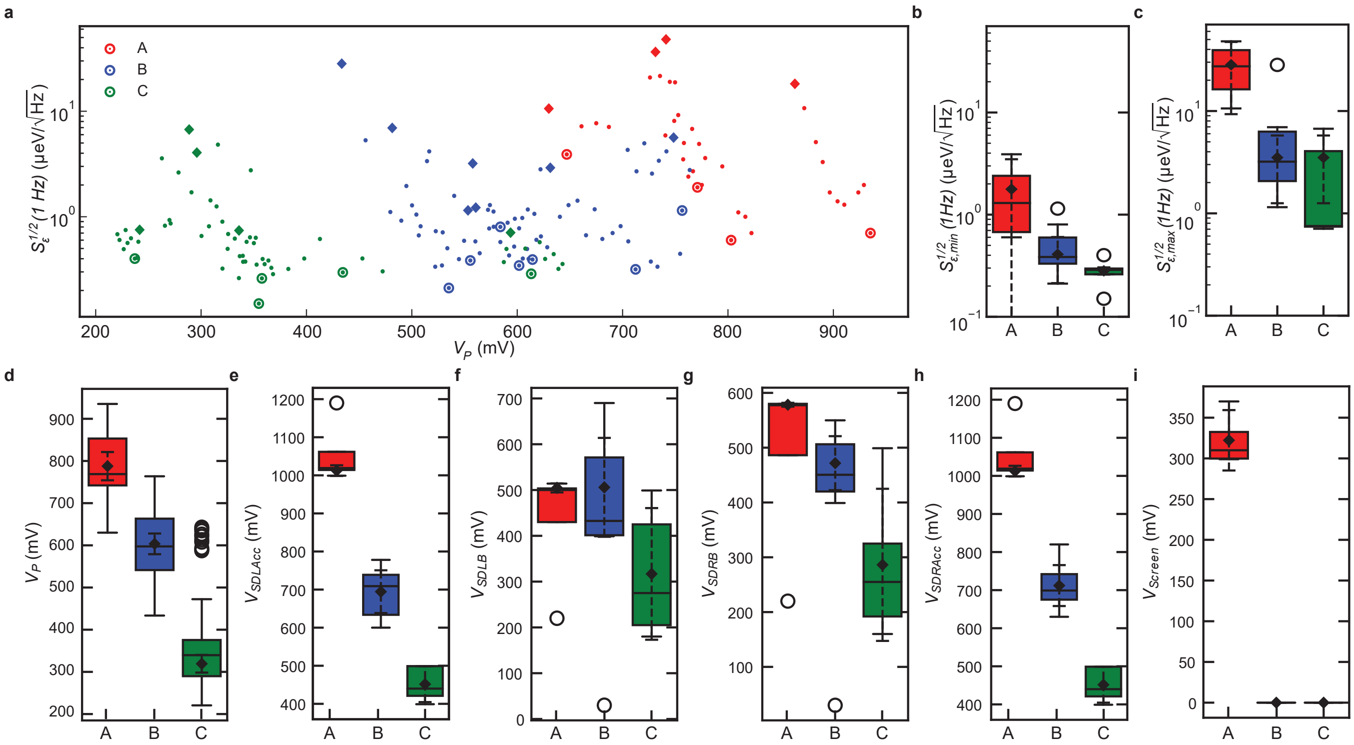

Figure S4. **a** Charge noise $S_\epsilon^{1/2}$ at 1 Hz as a function of the plunger gate voltage V_P for all measured devices of heterostructure A (red), B (blue), and C (green). Circles and diamonds highlight, respectively, the minimum ($S_{\epsilon,min}$) and maximum ($S_{\epsilon,max}$ charge noise at 1 Hz for each device upon varying V_P. For a given heterostructure, these $S_{\epsilon,min}$ and $S_{\epsilon,max}$ values build up the distributions plotted, respectively, in b (Fig. 2e main text) and c. The trend of charge noise improvement from A to B and C is observed both for $S_{E,min}$ and $S_{E,max}$. $S_{E,min}$ varies less than $S_{E,max}$ between different devices for a given heterostructure since $S_{E,max}$ is more affected by device-specific effects such as geometry of wave-function, screening, and the exact electron number on the island. Because we do not know the exact electron number, we believe that $S_{E,min}$ is more suited to compare between the different heterostructures.

d-i Distributions of the operation gate voltages of the plunger, SDLAcc, SDLB, SDRB, SDRAcc, and screening gates, respectively (see Fig. 1**f** in the main text) for heterostructure A (red, 4 devices measured), B (blue, 8 devices measured), and C (green, 5 devices measured). With the exception of gate SDLB, all operation voltages of the charge sensor are highest in heterostructure A and lowest in heterostructure C with a difference of up to 600 mV. Note that a global screening gate is only used for the operation of heterostructure A. Quartile box plots, mode (horizontal line), means (diamonds), 99% confidence intervals of the mean (dashed whiskers), and outliers (circles) are shown.

6. LEVER ARM EXTRACTION

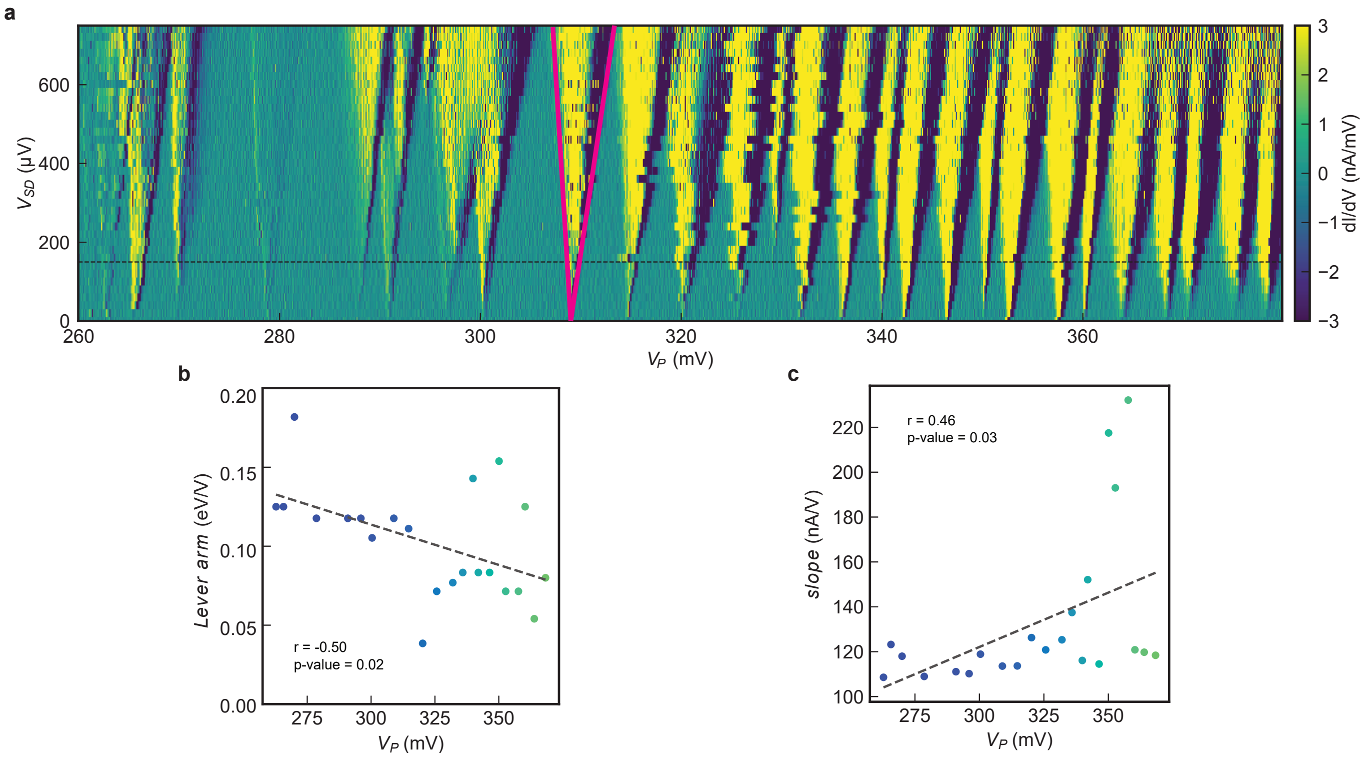

Figure S5. **a** Differential conductance (dI/dV) showing representative Coulomb blockade diamonds as a function of the source-drain voltage (V_{SD}) and plunger gate voltage (V_P) for heterostructure C. We derive the two slopes m_S and m_D on both sides of each Coulomb diamond. Using the equation $a = |\frac{m_S m_D}{m_S - m_D}|$, we extract a lever arm of $a = 0.12$ eV/V for the Coulomb peak at $V_P \approx 308$ mV, where we indicate m_S and m_D with magenta lines. The dashed line indicates the source-drain voltage ($V_{SD} = 150$ (μV)) used for the charge noise measurements. **b** Lever arm and **c** slope at the flank of the Coulomb peak for the peaks reported in **a**. We calculate the Pearson correlation coefficient (r), measuring the linear correlation between the two parameters. It varies between -1 and 1, with 0 implying no correlation. We also calculate the p-value of the null hypothesis, i.e., $r = 0$. We remember that the p-value indicates the probability of an uncorrelated system producing datasets that have a Pearson correlation at least as extreme as the one computed from these datasets. We remember that a p-value greater than 0.05 is considered not statistically relevant.

7. SIMULATIONS OF DEPHASING TIMES AND GATE FIDELITIES

Charge noise, as measured in this paper, leads to a loss of coherence for all kinds of quantum states. For qubit systems such decoherence can be described by two reference numbers: the qubit relaxation time T_1 and the qubit dephasing time T_2. Low frequency charge noise affects dominantly the dephasing time $T_2^\star$ of a qubit. Here, $T_2^\star$ references the free induction decay of a Ramsey experiment and describes the decay of a superposition state due to fluctuations in the resonance frequency of the qubit. The dephasing time $T_2^\star$ depends on the characteristics of the noise as well as the susceptibility of the qubit to the fluctuations. In short, charge qubits are more susceptible to charge noise than spin qubits. For a general qubit with energies $\mathcal{E}$ the dephasing time can be expressed as

$$T_2^\star = \frac{h}{\sqrt{2}\pi \left|\frac{\partial \mathcal{E}}{\partial \mu}\right| \sqrt{2\int_{f_{\rm lf}}^{f_{\rm hf}} S_\epsilon(f)df}}, \tag{8}$$

where S_ϵ is the measured noise spectral density of the chemical potential μ and $f_{\rm hf,(lf)}$ are the high (low-) frequency cut-off frequency. Note, that this simple expression for the dephasing time only holds away from a sweet spot [4, 5], $\frac{\partial \mathcal{E}}{\partial \mu} = 0$.

Dephasing of charge qubit

A charge qubit in general consist of two charge states with a difference in chemical potential $\epsilon = \mu_2 - \mu_1$ that are coupled via a tunnel matrix element t_c. Such a system can be described by the simple Hamiltonian

$$H = \frac{\epsilon}{2}\sigma_z + t_c\sigma_x, \tag{9}$$

where σ_x, σ_y, and σ_z are the three Pauli matrices. Charge noise couples to the charge qubit directly via their chemical potentials. As a consequence a charge qubit is maximally susceptible to charge noise and we find $\frac{\partial \mathcal{E}}{\partial \mu} = 1$ in the regime $\epsilon \gg t_c$. We take the values for the frequency cut-offs $f_{\mathrm{hf}} = 33\,\mathrm{GHz}$ from Ref. [6] and $f_{\mathrm{lf}} = 1.6\,\mathrm{mHz}$ for our simulation to ease comparison.

Dephasing of spin qubit

A spin qubit is ideally not affected by changes in the electrostatic environment from charge noise. However, due to intrinsic spin-orbit interaction (SOI) and artificial SOI through a micromagnet charge noise can couple to the spin degree of freedom. For a spin qubit made in SiGe using a micromagnet we find

$$\frac{\partial \mathcal{E}}{\partial \mu} = \alpha_{\mathrm{sensor}}\frac{\partial x}{\partial V}\frac{\partial B_z}{\partial x}\mu_B g = 1.6 \times 10^{-5}\frac{\partial \mathcal{E}}{\partial \mu} = \alpha_{\mathrm{sensor}}\frac{\partial x}{\partial V}\frac{\partial B_z}{\partial x}\mu_B g = 1.6 \times 10^{-5} \tag{10}$$

with the voltage displacement $\frac{\partial x}{\partial V} = 0.024\,\mathrm{nm/mV}$, field gradient $\frac{\partial B_z}{\partial x} = 0.08\,\mathrm{mT/nm}$, Bohr's Magneton $\mu_B = 0.0579\,\mu\mathrm{eV/mT}$, g-factor $g = 2$, lever arm $\alpha_{\mathrm{sensor}} = 0.07\,\mathrm{eV/V}$, and frequency cut-offs $f_{\mathrm{hf}} = 10\,\mathrm{kHz}$ and $f_{\mathrm{lf}} = 1.6\,\mathrm{mHz}$ all taken from Ref. [7].

Gate fidelity simulations

In order to extrapolate the performance of a two-qubit CZ gate from the measured charge noise we perform numerical simulations. The details of the simulations are described in Ref. [8] using the measured charge noise as an input. We simulate the unitary evolution operator of a CZ two-qubit gate using adiabatic barrier control at the detuning charge noise sweet spot. Colored charge noise is numerically generated using the Fourier filter method [9, 10] and added to the control pulses. For the simulation we use an additional heuristic lever arm $\alpha_{\mathrm{barrier}} = 1\,\mathrm{mV}/\mu\mathrm{eV}$ into consideration to translate the measured fluctuations in chemical potential to fluctuations in barrier voltage in the simulation. With this specification the charge noise measured in Ref. [8] would translate to $S_\epsilon^{1/2} = 0.4\,\mu\mathrm{eV/Hz}^{1/2}$, a reasonable assumption. To benchmark the performance we compute the average gate infidelity a commonly used metric for the quality of gates for all measured spectral densities $S_\epsilon(f) = S_\epsilon/f^\alpha$.

---

[1] E. J. Connors, J. Nelson, H. Qiao, L. F. Edge, and J. M. Nichol, Low-frequency charge noise in Si/SiGe quantum dots, Physical Review B **100**, 165305 (2019).

[2] M. Lodari, N. W. Hendrickx, W. I. L. Lawrie, T.-K. Hsiao, L. M. K. Vandersypen, A. Sammak, M. Veldhorst, and G. Scappucci, Low percolation density and charge noise with holes in germanium, Materials for Quantum Technology **1**, 011002 (2021).

[3] C. Spence, B. Cardoso-Paz, V. Michal, E. Chanrion, D. J. Niegemann, B. Jadot, P.-A. Mortemousque, B. Klemt, V. Thiney, B. Bertrand, L. Hutin, C. Bäuerle, F. Balestro, M. Vinet, Y.-M. Niquet, T. Meunier, and M. Urdampilleta, Probing charge noise in few electron CMOS quantum dots (2022), arXiv:2209.01853 [cond-mat].

[4] Y. Makhlin and A. Shnirman, Dephasing of Solid-State Qubits at Optimal Points, Phys. Rev. Lett. **92**, 178301 (2004).

[5] M. Russ and G. Burkard, Asymmetric resonant exchange qubit under the influence of electrical noise, Phys. Rev. B **91**, 235411 (2015).

[6] E. R. MacQuarrie, S. F. Neyens, J. P. Dodson, J. Corrigan, B. Thorgrimsson, N. Holman, M. Palma, L. F. Edge, M. Friesen, S. N. Coppersmith, and M. A. Eriksson, Progress toward a capacitively mediated cnot between two charge qubits in si/sige, npj Quantum Information **6**, 81 (2020).

[7] T. Struck, A. Hollmann, F. Schauer, O. Fedorets, A. Schmidbauer, K. Sawano, H. Riemann, N. V. Abrosimov, Łukasz Cywiński, D. Bougeard, and L. R. Schreiber, Low-frequency spin qubit energy splitting noise in highly purified 28si/sige, npj Quantum Information **6**, 40 (2020).

[8] X. Xue, M. Russ, N. Samkharadze, B. Undseth, A. Sammak, G. Scappucci, and L. M. K. Vandersypen, Quantum logic with spin qubits crossing the surface code threshold, Nature **601**, 343 (2022).

[9] Y.-C. Yang, S. N. Coppersmith, and M. Friesen, Achieving high-fidelity single-qubit gates in a strongly driven charge qubit with 1/f charge noise, npj Quantum Information **5**, 12 (2019).

[10] J. V. Koski, A. J. Landig, M. Russ, J. C. Abadillo-Uriel, P. Scarlino, B. Kratochwil, C. Reichl, W. Wegscheider, G. Burkard, M. Friesen, S. N. Coppersmith, A. Wallraff, K. Ensslin, and T. Ihn, Strong photon coupling to the quadrupole moment of an electron in a solid-state qubit, Nature Physics **16**, 642 (2020).
